# Terahertz Constant Velocity Flying Spot for 3D Tomographic Imaging

**DOI:** 10.3390/jimaging9060112

**Published:** 2023-05-31

**Authors:** Abderezak Aouali, Stéphane Chevalier, Alain Sommier, Christophe Pradere

**Affiliations:** 1 I2M TREFLE, UMR 5295 CNRS-UB-ENSAM, 351 Cours de la Libération, 33400 Talence, France; 2 EPSYL-Alcen, Esplanade des Arts et Métiers, CEDEX, 33405 Talence, France

**Keywords:** infrared thermospectroscopy, 3D tomography, terahertz imaging sensor, chemical information

## Abstract

This work reports on a terahertz tomography technique using constant velocity flying spot scanning as illumination. This technique is essentially based on the combination of a hyperspectral thermoconverter and an infrared camera used as a sensor, a source of terahertz radiation held on a translation scanner, and a vial of hydroalcoholic gel used as a sample and mounted on a rotating stage for the measurement of its absorbance at several angular positions. From the projections made in 2.5 h and expressed in terms of sinograms, the 3D volume of the absorption coefficient of the vial is reconstructed by a back-projection method based on the inverse Radon transform. This result confirms that this technique is usable on samples of complex and nonaxisymmetric shapes; moreover, it allows 3D qualitative chemical information with a possible phase separation in the terahertz spectral range to be obtained in heterogeneous and complex semitransparent media.

## 1. Introduction

Tomography is an imaging technique that allows the visualization of the 3D volume of an object from a series of 2D acquisitions. This technique is used in various fields, such as medicine, geology, and nondestructive testing. There are several acquisition techniques for performing tomography, for example: confocal tomography, reconstruction by angular projections (Radon), and laminography. This paper reports an experimental setup and inverse Radon transform processing methods to perform 3D tomography of heterogeneous and complex shape samples in the terahertz spectral range.

For many years, various works on volume reconstruction of samples in different spectral ranges have been carried out, such as the X-ray range [1,2], the visible range [3], and the infrared range [4,5]. This is because instrumentation and different tools (notably sources, spectroscopes, detectors, lenses) are available and well-developed for use in these spectral ranges. In contrast, few works on tomography in the terahertz spectral range have been reported [6]. However, interest in the various properties of THz radiation is increasing, and the low-energy and non-ionizing aspect of THz radiation opens up a wide range of applications based essentially on the particular spectroscopic properties of this radiation. There are therefore many applications of THz in various fields, for example, spectroscopy [7,8], astrophysics [9], security [10,11,12], nondestructive testing [13,14,15], biology [16,17], etc.

The current limitations of 3D imaging in the terahertz spectral range are multiple [18] and stem from the following facts. (i) The data acquisition time is long: it takes several minutes to several hours to develop an image. (ii) The imaging distance (distance between the sample and the sensor) is short, mainly due to the low power (except for free electron lasers) and the high divergence of the THz beam. (iii) The components for terahertz imaging (such as lenses) are poorly developed or unavailable. Wang et al. [19] carried out 3D terahertz transmission tomography on homogeneous objects using a THz source with a frequency of 278.6 GHz (λ=1.08mm) and a mono-spectral point sensor (Golay cell sensor with a diameter of 6 mm). The time required for an object of size 30×30mm is equal to 9 h. Balacey et al. [20] developed a 3D point-by-point THz imaging system in transmission using a 287 GHz frequency source and a spectrally limited point sensor (220–330 GHz diode). The time required to complete the acquisition of an object of size 148×120mm is equal to 5 h and 24 mn. Yim et al. [21] exploited the variable and large penetration depth of multispectral THz radiation coupled with mathematical processing (Hilbert transform) to implement a THz imaging system in reflection mode. The system is adapted for the analysis of small surfaces and allows 3D tomography of electronic chips (32×32 pixels) in 10 s. The primary challenge of our study consists of developing a fast 3D THz imaging system using a hyperspectral sensor and high-performance instrumentation and control. This system will therefore allow 3D imaging for the chemical analysis of heterogeneous samples as well as the study of dynamic physical phenomena.

Currently, there are several ways to generate THz radiation [22] as well as to detect it [23]. THz detectors can be classified into three families: thermal detectors (bolometers) [24], electro-optical detectors [25], and photoconductive detectors [24]. In this study, the sensor used is a hyperspectral thermoconverter [26] from the family of thermal detectors for the detection of THz; this thermoconverter is a thin and homogeneous carbon-based film. When a hyperspectral source optically illuminates it, this film absorbs the radiation and warms up by the photothermal effect (the absorption of the radiation depends on the illumination wavelength [27]; for example, 100% is absorbed from the visible to the far IR and only 61% is absorbed in the millimeter range). The heating of the film creates spatial thermal transfer and diffusion. Hence, the temperature variation of the thermoconverter can be measured with a thermal camera. Consequently, the combination of the thermoconverter-IR camera pair with a THz source results in a low-cost broadband imaging system that is easily adaptable in terms of size and configuration.

According to the previous literature review and to the authors’ best knowledge, few tomography-based studies have been reported in the terahertz spectral region. For this reason, it becomes important to develop new experimental tools to perform hyperspectral and fast tomography to study chemically heterogeneous objects and dynamic physical phenomena in this region. Thus, the present research represents a step towards this goal with the development of a new tomographic benchmark in the THz region using both a Midwave Infrared (MWIR) camera coupled to a thermoconverter and a THz source. In Section 1, the results obtained from the volume reconstruction of a heterogeneous object (hydroalcoholic gel vial) are illustrated; these results are then discussed and compared to tomographic reconstructions in the THz region found in the literature. In Section 2, the experimental setup and the methodology used for the 3D tomographic reconstruction are described.

## 2. Materials and Methods

### 2.1. Experimental Setup

**Figure 1:**
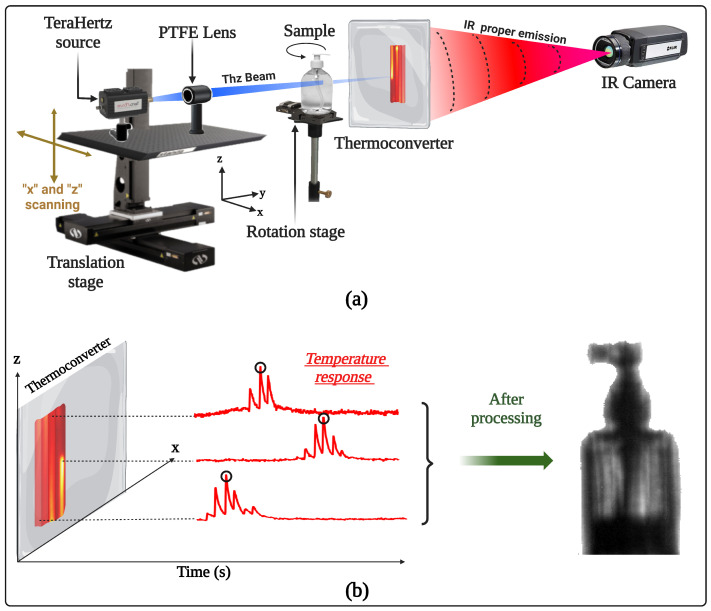
Experimental setup and a priori numerical processing of the acquisitions: (**a**) complete description of the experimental setup dedicated to 3D terahertz imaging; (**b**) schematic of the numerical processing necessary on each acquired film to obtain a useful image.

The experimental setup is described in Figure 1a. An electronic source from TERASENSE: IMPATT diodes, with a power of 400mW and a frequency of 100GHz, is used. The wavelength corresponding to the frequency of the source is equal to 3mm and therefore constitutes the theoretical limit of the spatial resolution of the measurements. Indeed, all objects smaller than 3mm will be subject to the effects of optical diffraction [28]. On the other hand, a beam of millimeter electromagnetic radiation is known to be divergent and difficult to manage optically, which implies the need to design an efficient opto-mechanical system that allows the scanning of an object with the smallest possible spot, thus providing a maximum signal. To remedy this problem, a Teflon (PTFE) lens with a focal length of 152mm and diameter of 50mm is used to focus the beam coming from the source.

The scanner+PTFE lens assembly is mounted on a stage, which is attached to a mechanical scanner. The scanner is composed of two translation motors (X-LRT-AEC Zaber Series) assembled to be able to scan in the *x* and *z* directions. The speed of the motors is 50mm/s, which allows us to scan a surface of 5cm×5cm with a focused beam of diameter equal to 6mm in 10 s. The sample is placed in front of the acquisition system and rests on a rotation motor (X-RST Zaber series) to collect the different angular projections. The acquisition system is composed of the coupled thermoconverter [26] as well as an infrared MCT camera (FLIR SC7000, 320×256 pixels, pitch size of 25 μm, spectral range between 9 and 11 μm) equipped with an infrared objective with a focal length of 25mm, allowing a spatial resolution of 200 μm per pixel. For each angular projection, the scanning time is 50 s, and the generated film size is 107 Mb. The sample to be scanned is placed (see Figure 1a) in the trajectory of the electromagnetic beam and before the thermoconverter at room temperature and humidity, which allows the use of ombroscopic imaging and takes into account the interactions of the electromagnetic beam with the components constituting the sample. This light–matter interaction leads to the measurement of intensive physical quantities such as absorbance or transmittance.

To obtain a useful image of the object scanned by this method, numerical processing of the acquired film is required. Figure 1b explains the principle of the processing applied using Matlab software, which consists of choosing the maximum of the signal recorded by each pixel over time. Indeed, this maximum corresponds to the passage of the THz beam over the pixel concerned or its nearest neighbors. This yields the advantage of reducing the effects of thermal diffusion and optical diffraction on the thermoconverter and thus benefiting from rapid processing. However, the quantitative aspect of the measurements is lost.

### 2.2. Measurement Procedure

**Figure 2:**
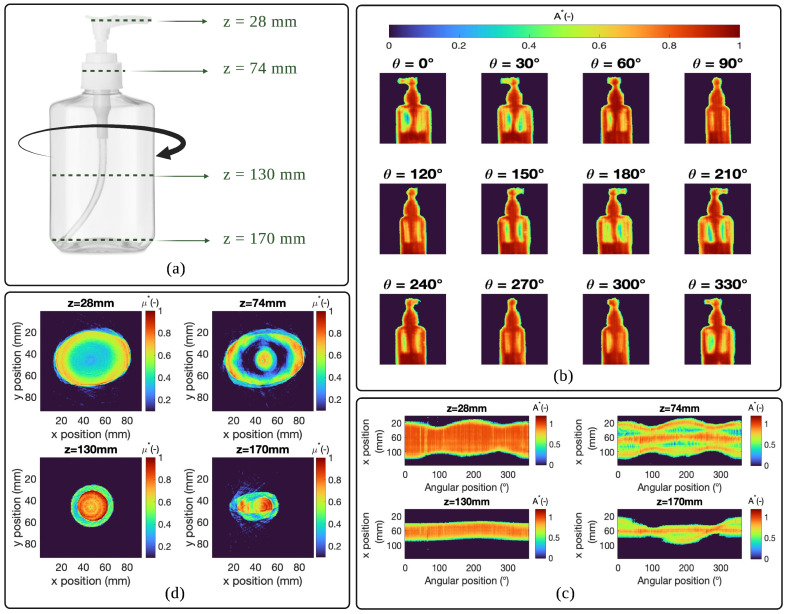
Measurement procedure and reconstruction of the sample sections: (**a**) visible image of the hydroalcoholic gel vial used; (**b**) images of the absorbance of the hydroalcoholic gel vial as a function of different rotation angles; (**c**) representation of the normalised sinograms at different “z” positions and as a function of the rotation angles; (**d**) representation of the normalized reconstructed sections at different “z” positions of the sample using the filtered back-projection method.

The sample used for this study is a hydroalcoholic gel vial with dimensions equal to 170mm×65mm×50mm. The choice of such a sample is motivated by the fact that it is heterogeneous and of complex shape (nonaxisymmetric). In fact, the nonaxisymmetric shape of the vial will allow the validation of the methodology as well as the experimental configuration proposed here. As previously explained, the vial is placed on a motorized rotating stage to acquire the different angular projections (see Figure 2a), and the illumination is provided by the focused THz beam from the source and motorized translation stages that allow the scanning of the vial surface. With this procedure, the transmittance of the vial at the source wavelength can be calculated. In fact, the transmitted signal, *I*, after the passage of the vial has the expression:

(1) $$I\left(x,z,\theta_{i},\lambda ,C,T\right)=\Gamma \left(x,z,\theta_{i},\lambda ,C,T\right)I_{0}\left(x,z,\lambda \right),$$

then:

(2) $$\Gamma \left(x,z,\theta_{i},\lambda ,C,T\right)=\exp\left[-\int_{0}^{Ly}\mu \left(x,z,y,\lambda ,C,T\right)dy\right]=\frac{I\left(x,z,\theta_{i},\lambda ,C,T\right)}{I_{0}\left(x,z,\lambda \right)}.$$

where $I\left(x,z,\theta_{i},\lambda ,C,T\right)\;\left(W\cdot m^{-2}\cdot {\mathrm{sr}}^{-1}\right)$ is the transmitted signal as a function of wavelength and object concentration or chemical composition. $\Gamma (x,z,\theta_{i},\lambda ,C)$ is the transmittance, $I_{0}\left(x,z,\lambda \right)\;\left(W\cdot m^{-2}\cdot {\mathrm{sr}}^{-1}\right)$ is the intensity of the beam alone (background), μ(x,z,y,λ,C)$\left(m^{-1}\cdot {\mathrm{mol}}^{-1}\cdot K^{-1}\right)$ is the absorption coefficient, which depends on the chemical composition, temperature and pressure of the sample, and $L_{y}$ (m) corresponds to the thickness of the sample in the *y* direction.

Figure 2b shows the different angular projections of the absorbance of the hydroalcoholic gel vial (passing through the Radon transformed space) at the wavelength of the THz source (3mm) obtained as a function of the different angles of rotation of the motor. Recall that transmittance and absorbance are related by the following relationship:

(3) $$A\left(x,z,\theta_{i},\lambda ,C,T\right)=-\log_{10}\left[\Gamma \left(x,z,\theta_{i},\lambda ,C,T\right)\right]=\int_{0}^{Ly}\mu \left(x,z,y,\lambda ,C,T\right)dy$$

where *A* represents the absorbance of the vial at the source wavelength. The set of acquisitions of each line of the Radon transform (angular projections) obtained for rotation between 0 and 2π is called a sinogram. Figure 2c shows the representation of sinograms normalized with respect to the maximum for the different positions in the z-axis and as a function of the rotation angles. These sinograms are calculated from the angular projections of the absorbance.

One sinogram allows the reconstruction of a slice of the object based on the theme of the central slice. Figure 2d shows slices reconstructed from sinograms calculated previously using the filtered back-projection method. This method allows us to perform the inverse Radon transform and is essentially based on the application of an operator defined as follows:

(4) $$B\left[\mu \left(x,z,y,\lambda ,C\right)\right]=\int_{0}^{\pi }\tilde{A}\left(x\cos\theta +z\sin\theta ,\theta ,\lambda ,C\right)d\theta$$

where $\tilde{A}$ designates the Fourier transform in the polar coordinate of absorbance A. Finally, the 3D absorptivity field is reconstructed using Equation (5):

(5) $$\mu \left(x,z,y,\lambda ,C,T\right)=B\left\{F^{-1}[F\left[\tilde{A}\left(x,z,\theta ,\lambda ,C,T\right)\right]\cdot \left|W|\right]\right\}$$

where B represents the back-projection filter, F represents the Fourier transform, and W represents the ramp filter (Appendix A). By analyzing Equation (5), it can be determined that the sections reconstructed using the back-projection method physically represent the normalized absorption coefficient of the vial. This method allows access to an intrinsic quantity (absorption coefficient) and therefore to the qualitative chemical information with a phase separation of the samples.

## 3. Results and Discussion

In summary, it has been demonstrated that infrared thermography using an IR camera coupled to a deported bolometer allows for a low-cost broadband detection system. This detection system, when associated with an optimized experimental configuration, allows for 3D multispectral imaging. In fact, the exploitation of the multispectral aspect of the thermoconverter [27] allows spectroscopic imaging with a very wide spectral band with the same system (changing only the excitation source). Thus, this configuration makes it possible to achieve qualitative characterization and chemical analysis of heterogeneous objects.

**Figure 3:**
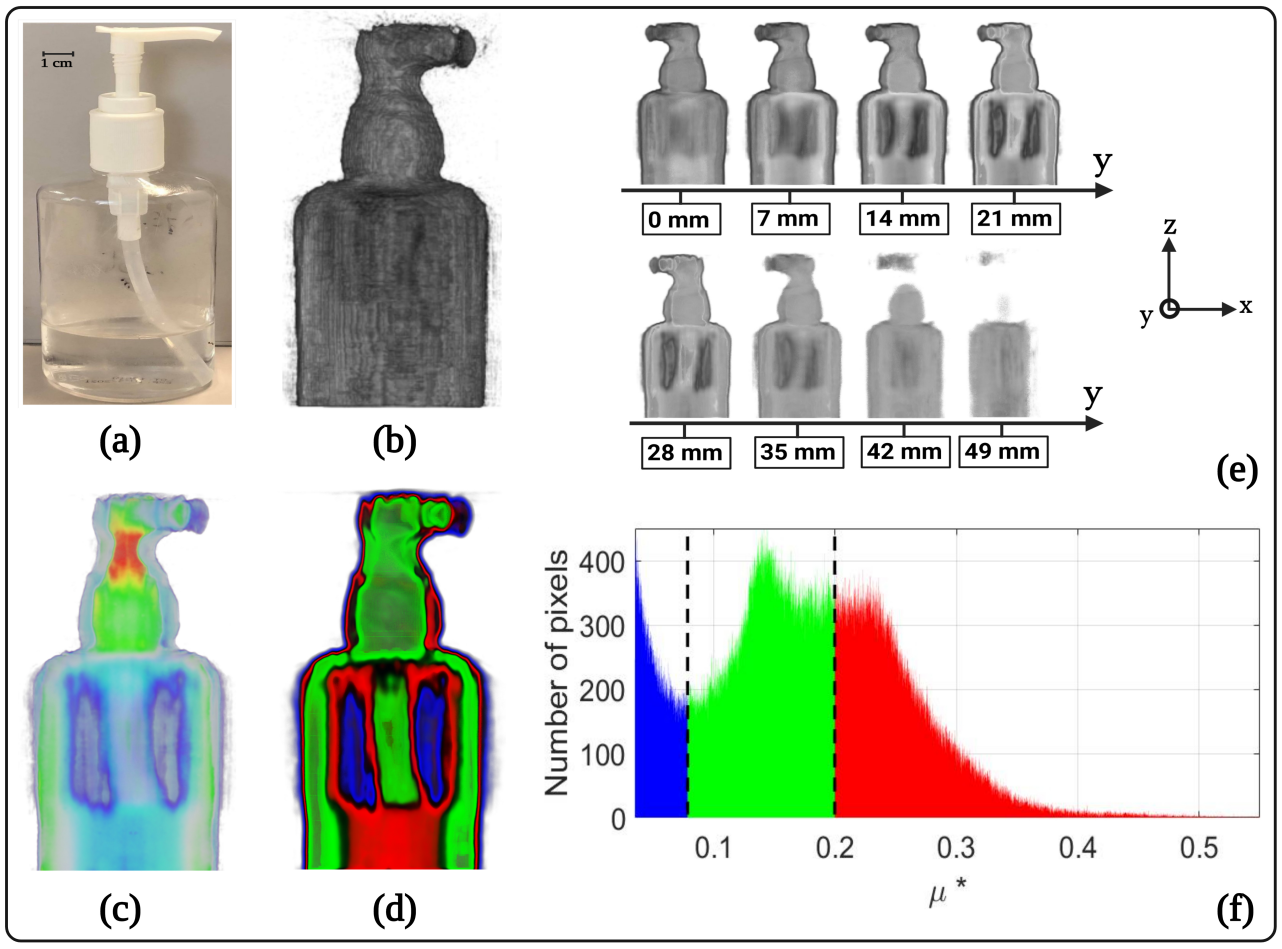
3D terahertz tomography of the normalised absorption coefficient obtained using a hydroalcoholic gel vial (half-filled with hydroalcoholic gel) as a sample: (**a**) photograph of the hydroalcoholic gel vial in the visible light; (**b**) external perspective view of the 3D absorption coefficient of the vial; (**c**) perspective view from the midplane of the 3D absorption coefficient of the vial; (**d**) perspective view from the midplane of the 3D absorption coefficient of the vial with segmentation of the absorption levels; (**e**) representation of different internal planes of the 3D absorption coefficient by scanning along the *y*-axis; (**f**) histogram obtained from the midplane of the segmented 3D absorption coefficient of the vial.

Figure 3 shows the 3D tomography of the absorption coefficient results obtained using the hydroalcoholic gel vial (filled to 35mm with hydroalcoholic gel of density equal to 0.862 Kg/m^3^) as a sample. The 3D tomography obtained in this study is normalized with respect to the maximum and allows only qualitative studies. To determine a quantitative aspect, a calibration of the absorbance based on known samples is necessary beforehand. Figure 3a shows the sample in the visible region. The heterogeneity due to the difference in the chemical nature of the components constituting the vial (solid plastic, liquid hydroalcoholic gel, gaseous ambient air) will allow us to highlight an absorbance contrast on the tomographic reconstructions. Figure 3b shows an external perspective view of the absorption coefficient of the vial reconstructed in 3D. Figure 3c,d show a perspective view from the midplane of the absorption coefficient of the vial reconstructed in 3D and the same view with segmentation of the absorption coefficient levels, respectively. The contrast in absorbance between the different media constituting the sample can thus be observed directly: air, hydroalcoholic gel and plastic. Figure 3f allows visualization of the histogram of the segmented plane and affirms that the air is the medium that absorbs the least ($\mu^{*}$ between 0 and 0.07), followed by the plastic ($\mu^{*}$ between 0.07 and 0.2), and finally the hydroalcoholic gel, which is the component that absorbs the most ($\mu^{*}$ between 0.2 and 0.5). This result was predictable because the hydroalcoholic gel is essentially composed of water, which is almost opaque at the wavelength of the source (λ=3mm) [29]. Finally, Figure 3e represents a scan of different planes of the reconstructed volume along the *y*-axis.

The spatial resolution of each 2D angular projection image is equal to 200μm and corresponds to the pixel size of the thermoconverter-IR camera combination. This result may seem surprising since the spatial resolution is much lower than the diffraction limit (which is conditioned by the illumination wavelength 3 mm). This can be explained by the use of the thermoconverter, which acts as a projection screen, and the acquisitions are made by the IR camera. In other words, this acquisition system aims to perform ombroscopic imaging with the resolution limit dictated by the IR camera [30,31]. If we interpret this result physically, the spatial resolution obtained here does not mean that this system allows for sub-wavelength imaging, but rather that the shadows of the sample are projected onto a much better spatially resolved screen.

**Figure 4:**
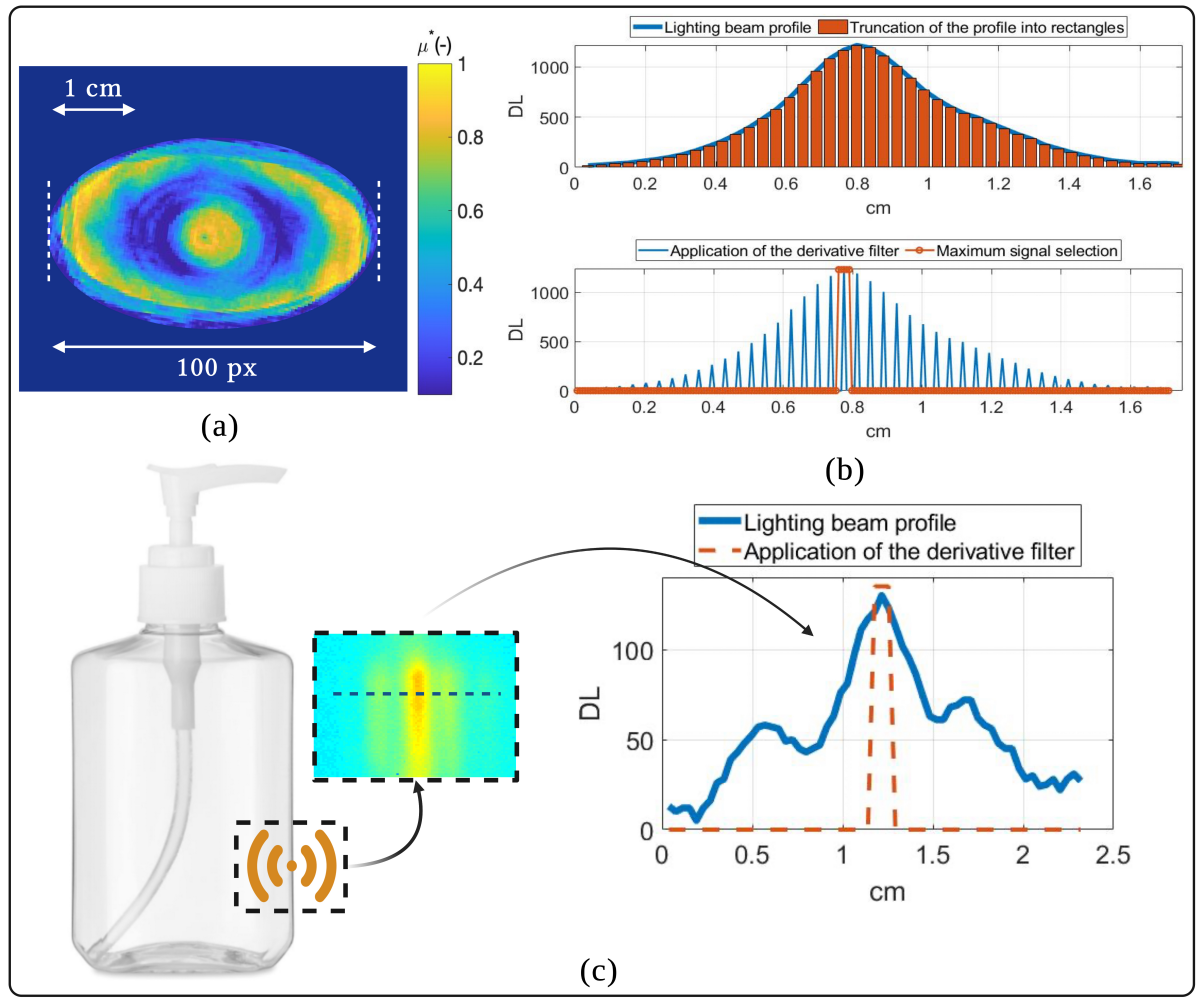
Spatial effects of the applied imaging method: (**a**) spatial resolution on a reconstructed section of the vial; (**b**) spatial derivation applied to the Gaussian-shaped THz beam; (**c**) effect of the spatial derivative filter in artificially eliminating optical diffraction.

The qualitative tomography obtained from the 2D angular projections is visually of very good quality, taking into account the wavelength of the illumination source. The spatial resolution of 3D tomography is usually expressed by the volume of the voxel (cubic pixel), the volume of the reconstructed voxel is clearly dependent on the experimental setup (pixel size of the thermoconverter-IR camera combination) as well as on the reconstruction method and algorithm (back-projection method). In our case, this volume was calculated experimentally by applying the ombroscopic imaging method on cylindrical metal rods of different diameters and known fixed height (Appendix B), so the volume of a voxel can be calculated and is equal to $V_{voxel}\;=\;0.06\;{\mathrm{mm}}^{3}$ (see Figure 4a).

The reconstructed 3D tomography of the absorption coefficient of the vial in this study required an acquisition and processing time of 2 h and 30 min for 180 angular projections of a surface equal to 220mm×120mm (50 s/projection), which is 2 times shorter than [20] and 3.5 times shorter than [19], which uses the same 3D reconstruction method with only 36 angular projections. Thus, with the same conditions as these works cited, i.e., an acquisition of only 36 angular projections, the time necessary to carry out 3D tomography with our system will be lowered to 30 min but the quality of the reconstruction will be affected, a factor of 5 times shorter than what is currently done (and 11 times shorter than [20] and 18 times shorter than [19]). This time gain is mainly due to the use of constant velocity flying spot illumination using two translation motors. This type of illumination provides a very good signal-to-noise ratio (equal to 10 in our case), but requires numerical processing of the acquired film to produce a useful image (one image produced for each film recorded). In our case, this treatment consists of choosing the maximum signal recorded by each pixel over time (temporal analysis). On the other hand, the spatial analogy of this temporal processing is a spatial derivative applied to the Gaussian-shaped THz beam, as shown in Figure 4b. Thus, this processing allows us, thanks to this spatial derivative action, to avoid optical diffraction, as shown in Figure 4c. Optical diffraction is a physical phenomenon related to the wave properties of light. In imaging, it essentially occurs when optical radiation encounters an obstacle smaller than the wavelength of that radiation. Taking into account the importance of the wavelength of the THz source used here “λ=3mm”, it is therefore logical that optical diffraction effects are accentuated when performing imaging with such a source. This result is therefore the first THz tomography (3D mapping of the absorption coefficient) of a heterogeneous object (liquid–air–solid) obtained using an original low-cost system (thermoconverter-IR camera coupling) that relies on thermographic measurements.

## 4. Conclusions

To conclude, in this work, a tomography technique in the terahertz spectral range has been developed to reconstruct the 3D absorption coefficient field in semitransparent THz media. In this paper, the methodology and the conception of the imaging system have been described, the most important point being the introduction of a hyperspectral thermoconverter coupled to an IR camera used as a sensor of THz radiation. This technique allowed us, using the inverse Radon transform, to reconstruct 3D volume cartography of the absorption coefficient of a vial of hydroalcoholic gel using THz radiation (λ=3mm). The time required for data acquisition and volume reconstruction was 2.5 h, and the volume spatial resolution was equal to $V_{voxel}\;=\;0.06\;{\mathrm{mm}}^{3}$. This new THz volume tomography method thus exhibits the ability to qualitatively reconstruct the volume of physical quantities related to the chemical composition of complex shaped and spectrally heterogeneous samples. This approach allows the generation of data cubes that represent three million voxels in an average time of two hours. Therefore, this research opens new perspectives in the field of THz imaging spectroscopy, as well as in the fields of hypercube and big data processing, with many applications expected in the fields of microfluidics, biology and living tissue.

## Figures and Tables

**Figure A1:**
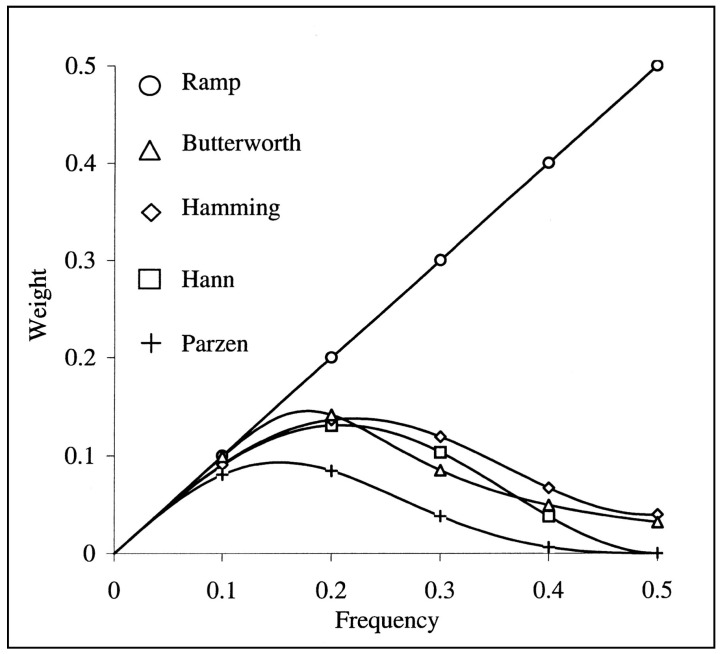
The different filters used in the filtered back-projection method [32].

**Figure A2:**
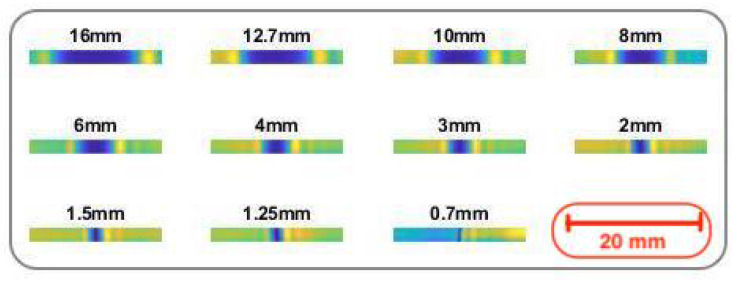
Measurements obtained for different metal rods.

**Figure A3:**
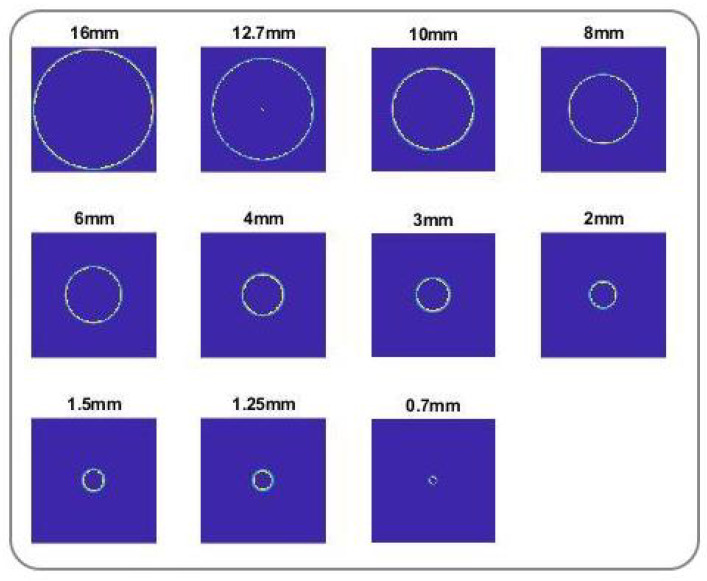
Slice reconstructed for each rod.

**Figure A4:**
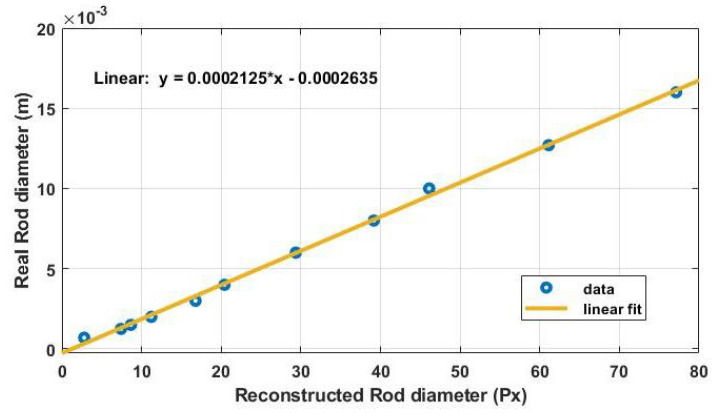
Diameter reconstructed in pixels according to the measured one.

## Data Availability

The data presented in this study are available on request from the corresponding author.

## Abbreviations

The following abbreviations are used in this manuscript:

2D, 3D	Two-dimensional, three-dimensional
C	Concentration
GHz	GigaHertz
IR	InfraRed
THz	TeraHertz
T	Temperature
θ	Angle
λ	Wavelength

## Appendix A. Ramp Filter

The ramp filter used in the back-projection method is a high-pass-type filter whose impulse response is given in Figure A1. The mathematical expression of this filter is given by the following equation:

(A1) $$W\left(\nu \right)=\left|\nu \right|$$

## Appendix B. Spatial Resolution and Resolution Limit

This part explains the approach used to experimentally measure the spatial resolution as well as to determine the resolution limit of our THz imaging system. This approach consists in applying the method of acquisition and 3D reconstruction on a set of cylindrical metal rods (axisymmetric) of different diameters and of known fixed height. Figure A2 shows an angular projection obtained by the acquisition system for each rod.

Figure A3 shows a reconstructed cross-section for each rod, if we relate the reconstructed cylinder diameter in pixels to the measured one as shown in Figure A4. A linear relationship is obtained, where the slope of the line represents the spatial resolution of the reconstruction. Furthermore, it can be seen that we always obtain an image of rods whose diameter is much smaller than the wavelength. It is therefore concluded that the resolution limit of the imaging system is given directly by the size of a pixel on the thermoconverter-IR camera pair. This is due to the fact that we are imaging ombroscopically on a screen (thermoconverter) and exploiting a temporal thermal response for data processing that allows us to remove spatial artifacts (mainly diffraction).
